# Modern thromboprophylaxis protocol based on guidelines applied in a respiratory intensive care unit: a single-center prospective cohort study

**DOI:** 10.1186/s12959-022-00439-2

**Published:** 2022-12-12

**Authors:** Xiao Tang, Wen-Rui Lyu, Yu Jin, Rui Wang, Xu-Yan Li, Ying Li, Chun-Yan Zhang, Wei Zhao, Zhao-Hui Tong, Bing Sun

**Affiliations:** 1 Department of Respiratory and Critical Care Medicine, Beijing Institute of Respiratory Medicine and Beijing Chao-Yang Hospital, Capital Medical University, No. 8 Gongtinan Road, Chaoyang, Beijing, 100020 China; 2 Department of Ultrasonic diagnosis, Beijing Chao-Yang Hospital, Capital Medical University, Beijing, China

**Keywords:** Venous thromboembolism, Thromboprophylaxis, Critical illness, Intensive care unit, Risk factors

## Abstract

**Background:**

Critically ill patients in intensive care units (ICUs) are at high risk of venous thromboembolism (VTE). This study aimed to explore the prophylaxis effect under a guideline-based thromboprophylaxis protocol among critically ill patients in a respiratory ICU.

**Methods:**

For this single-center prospective cohort study, we followed the thromboprophylaxis protocol, which was drawn up based on relevant guidelines and Chinese experts’ advice. Clinical data were entered into an electronic case report form and analyzed. Multivariate logistic regression was conducted to explore independent risk factors of VTE event under this protocol.

**Results:**

From August 1, 2014, to December 31, 2020, 884 patients underwent thromboprophylaxis according to this protocol; 10.5% of them received mechanical prophylaxis, 43.8% received pharmacological prophylaxis, and 45.7% received pharmacological combined with mechanical prophylaxis. The proportion of VTE events was 14.3% for patients who received the thromboprophylaxis protocol, of which 0.1% had pulmonary thromboembolism (PTE), 2.0% had proximal deep vein thrombosis (DVT), and 12.1% had isolated distal DVT. There was no significant difference between different thromboprophylaxis measures. Cirrhosis (OR 5.789, 95% CI [1.402, 23.894], *P* = 0.015), acute asthma exacerbation (OR 39.999, 95% CI [4.704, 340.083], *P* = 0.001), and extracorporeal membrane oxygenation treatment (OR 22.237, 95%CI [4.824, 102.502], *P* < 0.001) were independent risk factors for proximal DVT under thromboprophylaxis.

**Conclusions:**

The thromboprophylaxis protocol based on guidelines applied in the ICU was practicable and could help decrease the proportion of PTE and proximal DVT events. The risk factors of VTE events happening under the thromboprophylaxis protocol require more attention.

**Trial registration:**

ClinicalTrials.gov: NCT02213978.

**Supplementary Information:**

The online version contains supplementary material available at 10.1186/s12959-022-00439-2.

## Background

Venous thromboembolism (VTE) comprises pulmonary thromboembolism (PTE) and deep vein thrombosis (DVT), with an annual proportion ranging from 0.75 to 2.69 per 1000 people among the populations of Europe and North America [1]. Previous research reported that the age and sex-adjusted proportion of VTE among China’s population increased from 3.2 to 17.5 per 100,000 people from 2007 to 2016 [2]. Under appropriate prophylaxis measures, the proportion of VTE for inpatients has been found to decrease from about 4.9–14.9% to 2.7–5.5% [3, 4]. Patients with critical illness are at high risk of VTE [5, 6]. A previous study showed that the rates of DVT ranged from 13 to 31% in critically ill patients without prophylaxis measures [7]. Moreover, the frequency of VTE in patients in the intensive care unit (ICU) receiving thromboprophylaxis ranged from 5.1 to 15.5% [8]. Many studies have also proposed that VTE events are associated with a poor prognosis in critically ill patients [9, 10].

According to the present guidelines, thromboprophylaxis practice should be done on the basis of a VTE risk evaluation [5, 6]. However, a multinational cross-sectional study reported that 51.8% of hospitalized patients were at risk of VTE, with only one half of those receiving prophylaxis complying with related guidelines [11]. A previous study showed that the overall rate of guidelines-recommended prophylactic method was 10.3% in surgical and medical patients [12]. Meanwhile, the current status of VTE prophylaxis in ICUs is also not optimistic [9, 13]. Our previous study suggested that the awareness rate of VTE prophylaxis among the medical staff of ICUs in North China remains limited, which may lead to a lack of standardized VTE prophylaxis [14]. However, the proportion of major bleeding in critically ill patients under heparin thromboprophylaxis has been found to be about 4–6% [15, 16], which may limit VTE prophylaxis practice in ICUs.

Since August 2014, a thromboprophylaxis protocol has been applied in the respiratory ICU of Beijing Chao-Yang Hospital. This protocol was drawn up based on the relevant guideline and experts’ advice [5, 6] and considers the specialty of the respiratory ICU. This study aimed to explore the effects of VTE prophylaxis on the proportion of VTE, and the risk factors of VTE among critically ill patients in the ICU after receiving the thromboprophylaxis protocol.

## Methods

### Study design and patients

This study was a single-center, prospective cohort study. Patients admitted to the respiratory ICU from August 1, 2014, to December 31, 2020, were enrolled in this study. Patients aged 18 or older with a length of stay (LOS) in the ICU of more than 48 hours were included in this study. The exclusion criteria were admission to the ICU because of acute PTE and/or proximal DVT event, readmission in 48 hours after transferring out of the ICU, and refusal to participate in the study. This study was reviewed and approved by the Ethics Committee of Beijing Chao-Yang Hospital (2014-Ke-142). Informed consent was obtained from the patients or their legal guardian.

### Thromboprophylaxis protocol

**Fig. 1:**
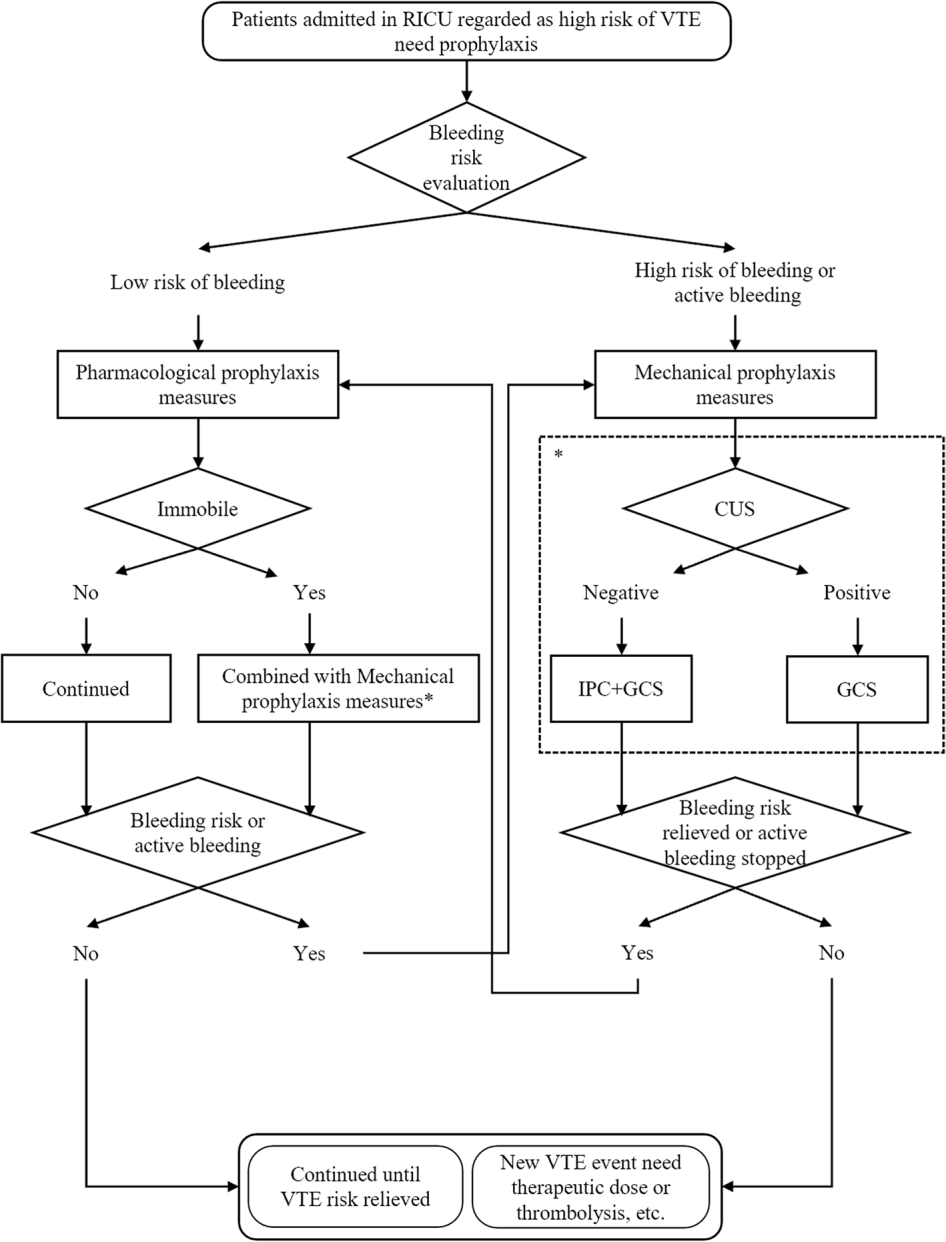
Thromboprophylaxis protocol. CUS, compression ultrasonography; GCS, graduated compression stocking; IPC, intermittent pneumatic compression; VTE, venous thromboembolism

The thromboprophylaxis protocol was conducted in a 16-bed respiratory ICU. All critically ill patients in the respiratory ICU were considered as being at high risk of VTE and should receive VTE prophylaxis. Bleeding risk was evaluated first. If the patients had a high bleeding risk or already had active bleeding, mechanical prophylaxis measures were applied. Pharmacological prophylaxis measures were used on patients with a low bleeding risk. If these patients were immobile, such as in deep sedation, on a neuromuscular blocker, or paralyzed, they were stratified as being at extremely high risk of VTE, and pharmacological prophylaxis combined with mechanical measures were conducted. While the patients acquired active bleeding or bleeding risk increased during pharmacological prophylaxis, mechanical measures would be switched instead of pharmacological measures. The risk of bleeding or active bleeding needed dynamic evaluation, and pharmacological prophylaxis had to replace mechanical prophylaxis once the bleeding risk was relieved or active bleeding stopped. Before mechanical prophylaxis was implemented, compression ultrasonography (CUS) of lower extremity had to be conducted. Intermittent pneumatic compression (IPC) was not used while DVT existed; otherwise, IPC combined with graduated compression stocking (GSC) was used. The thromboprophylaxis protocol was maintained until the VTE risk was relieved or there was a new occurrence of VTE events that required therapeutic anticoagulation or thrombolysis (Fig. 1).

For the aspect of pharmacological prophylaxis, low molecular weight heparin (LMWH) with a prophylactic dose was the first choice in patients without contraindications. Enoxaparine with the dose of 40 mg subcutaneous injection per day was chosen for pharmacological prophylaxis. The dosage would be adjusted if the patients with extreme weight. Unfractionated heparin (UFH) was used as 5000 IU subcutaneous injection twice per day or continuous intravenous infusion with range of APTT about 1.2 times baseline was the alternative anticoagulant in patients with a non-bleeding contraindication of LMWH. If patients had a history of heparin-induced thrombocytopenia (HIT) or antithrombin deficiency, a prophylactic dose of a non-heparin anticoagulant was used, such as fondaparinux with 2.5 mg subcutaneous injection per day. If the patients had specific medical histories such as atrial fibrillation, chronic thromboembolic pulmonary hypertension, or post-cardiac surgery, the dosage of anticoagulation had to be according to the disease-specific treatment need. The patients underwent extracorporeal support including extracorporeal membrane oxygenation (ECMO) or continuous renal replacement therapy (CRRT), and the anticoagulation management had to be in line with the relevant protocol.

If patients with an active gastroduodenal ulcer, prior bleeding history in the 3 months before admission, low platelet count (less than 5 × 10^9^/L), hepatic failure (international normalized ratio higher than 1.5), and activated partial thromboplastin time (APTT) increased (10 s increased) without an anticoagulation agent, they were assessed as being at high risk of hemorrhage [16, 17].

The CUS examination was conducted as a screening of DVT in the first 24 hours after admission to ICU. During ICU hospitalization, if the patients presented with suspicious clinical manifestations of PTE or DVT, diagnosis and treatment process had to be according to the related guidelines [18]. If patients did not show any suspicious clinical symptoms, CUS was re-examined to avoid missing asymptomatic DVT before discharge from the ICU or death. DVT events referred to newly formed sites of lower extremity DVT.

### Outcomes

The primary outcome was newly developed VTE events during the ICU stay. Secondary outcomes comprised bleeding events, thrombocytopenia, all-cause mortality in the ICU, and length of ICU stay. Bleeding events included gastrointestinal bleeding, urinary tract bleeding, oral or nasal bleeding, lower respiratory tract bleeding, retroperitoneal bleeding, skin bleeding, intracranial bleeding, surgical incision bleeding, and vaginal bleeding. Major bleeding events were defined as hemoglobin decline ≥2 g/L, hemorrhage treated by blood transfusion of more than 2 units of red blood cells, retroperitoneal hemorrhage, intracranial hemorrhage, hemorrhagic shock, and fatal hemorrhage [19]. Thrombocytopenia was defined as a 30–50% reduction in the baseline platelet level [20]. Thrombocytopenia was recorded at any time during thromboprophylaxis and was analyzed to ascertain the causes by physicians [21–23]. If any anticoagulant-related thrombocytopenia occurred, the suspicious drug was ceased.

### Clinical data collection

Demographic and clinical data of the patients were entered into an electronic case report form and included the following: demographic characteristics (age and sex), diagnosis, comorbidities, complications, laboratory tests (e.g., routine blood test, coagulation function, liver function, renal function), and organ support. The Caprini score [24] and Padua score [5] were recorded during admission. The VTE prophylaxis measures, proportion of VTE and bleeding events, ICU mortality, and length of ICU stay were also documented.

### Statistics analysis

Statistical analysis was performed with IBM SPSS 26.0. Categorical variables were described as frequency (percentage), and differences between groups were tested by the Chi-square test and Fisher’s exact test. Continuous variables were described by the median (interquartile range [IQR]) because of the non-normality distribution. Differences between groups were tested by the Mann–Whitney U test or Kruskal–Wallis H test. Univariate and multivariate logistics regression analyses were conducted to explore the risk factors of VTE events despite being under this protocol. The multivariable regression model was adjusted for the following characteristics: age, D-dimer, blood component infusion, shock, LOS in ICU, CRRT, artificial airway, and cessation of thromboprophylaxis. Variables with *P* < 0.1 in univariate analysis were included in multivariable logistic regression analysis. *P* < 0.05 was considered statistically significant.

## Results

### Patients’ characteristics and VTE prophylaxis

**Fig. 2:**
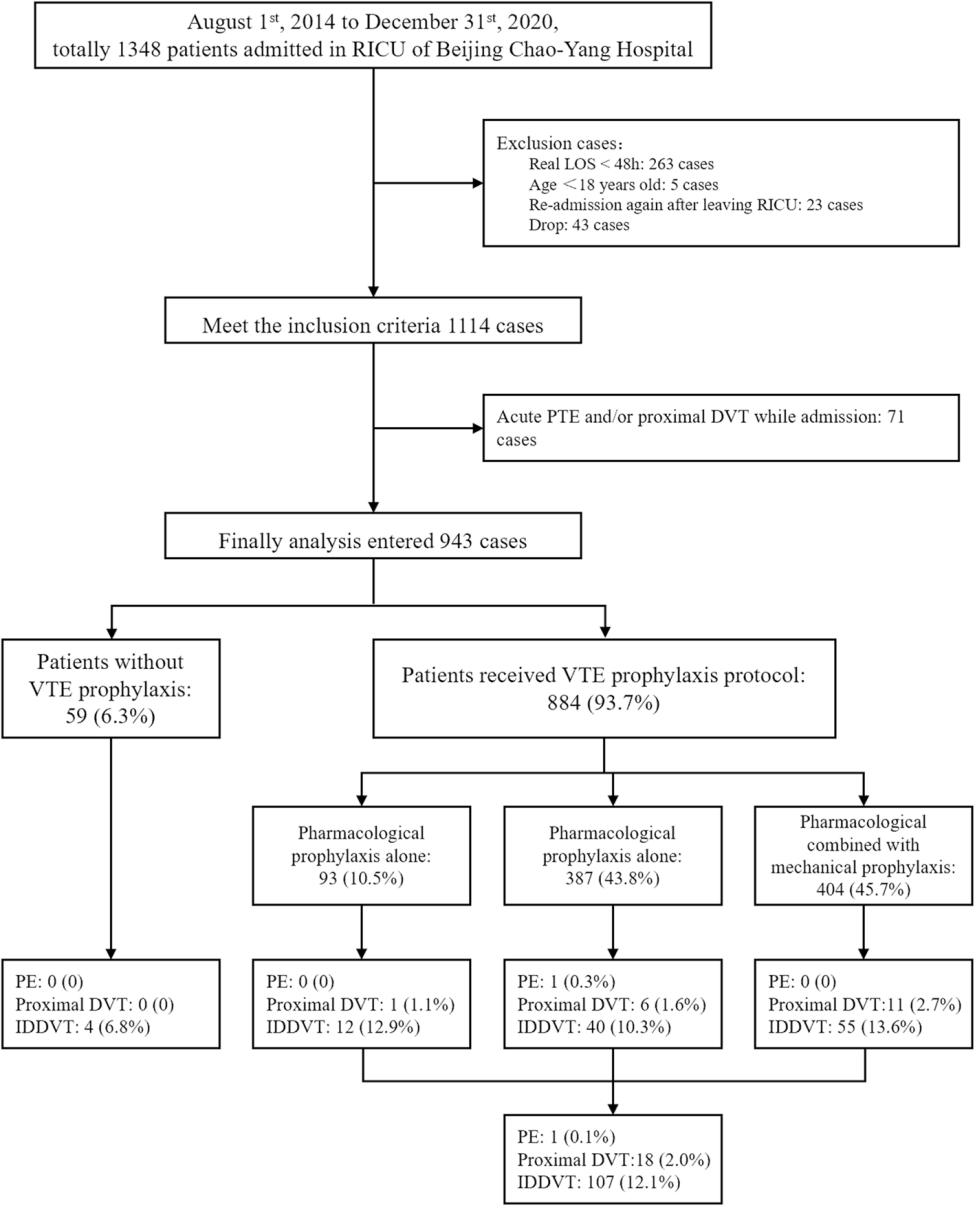
Flow chart. DVT, deep vein thrombosis; LOS, length of stay; IDDVT, isolated distal deep vein thrombosis; PTE, pulmonary thromboembolism; RICU, respiratory intensive care unit; VTE, venous thromboembolism

From August 1, 2014, to December 31, 2020, there were 1057 patients admitted to the respiratory ICU. Overall, a total of 943 cases were ultimately screened in the study (Fig. 2). Of these, 59 (6.3%) cases did not undergo any VTE prophylaxis (Supplementary Table 1). Finally, 884 patients received the thromboprophylaxis protocol, with the rate of VTE prophylaxis being 93.7%. Furthermore, 10.5% of patients received mechanical prophylaxis, 43.8% underwent pharmacological prophylaxis, and 45.7% received pharmacological prophylaxis combined with mechanical prophylaxis (Table 1).

**Table 1:** Characteristics of patients in different prophylaxis groups

	Overall (*N* = 884)	Mechanical prophylaxis group (*N* = 93)	Pharmacological prophylaxis group (*N* = 387)	Pharmacological combined mechanical prophylaxis group (*N* = 404)	*P*-value
Age (years, IQR)	61 (48, 70)	59 (46, 70)	62 (50, 73)	61 (47, 69)	0.074
≥ 70 years, n (%)	250 (28.3)	24 (25.8)	127 (32.8) †	99 (24.5) †	0.029
Male, n (%)	591 (66.9)	64 (68.8)	252 (65.1)	275 (68.1)	0.626
BMI (kg/m^2^, IQR)	23.7 (20.8, 26.7)	23.7 (21.3, 26.7)	23.4 (20.5, 26.7)	23.9 (21.2, 26.7)	0.402
BMI ≥ 30 kg/m^2^, n (%)	84 (9.7)	11 (11.8)	35 (9.2)	38 (9.6)	0.742
Underlying disease, n (%)					
Hypertension	366 (41.4)	44 (47.3)	157 (40.6)	165 (40.8)	0.478
Diabetes mellitus	192 (21.7)	28 (30.1)	78 (20.2)	86 (21.3)	0.109
Coronary artery disease	112 (12.7)	9 (9.7)	56 (14.5)	47 (11.6)	0.319
Chronic heart failure	125 (14.1)	6 (6.5)	56 (14.5)	63 (15.6)	0.071
Chronic renal dysfunction	78 (8.8)	9 (9.7)	26 (6.7)	43 (10.6)	0.146
Hepatic failure due to cirrhosis	43 (4.9)	3 (3.2)	13 (3.4)	27 (6.7)	0.084
Solid-organ malignancy	58 (6.6)	7 (7.5)	21 (5.4)	30 (7.4)	0.503
VTE history	25 (2.8)	3 (3.2)	8 (2.1)	14 (3.5)	0.444
Cause of admission, n (%)					
ARDS	274 (31.0)	18 (19.4)	109 (28.2)	147 (36.4)*	0.002
Pneumonia	593 (67.1)	65 (69.9)	234 (60.5)†	294 (72.8)†	0.001
IPA	25 (2.8)	2 (2.2)	18 (4.7)†	5 (1.2)†	0.012
AECOPD	101 (11.4)	3 (3.2)	60 (15.5)*	38 (9.4)	0.001
Acute exacerbation of asthma	17 (1.9)	0 (0)	12 (3.1)	5 (1.2)	0.075
Sepsis	73 (8.3)	10 (10.8)	24 (6.2)	39 (9.7)	0.137
Post-surgery	90 (10.2)	13 (14.0)	32 (8.3)	45 (11.1)	0.182
Caprini score, n%					
High or extremely high risk (≥3)	788 (89.2)	82 (88.2)	342 (88.6)	364 (90.1)	0.746
Padua score (High risk), n (%)	306 (34.7)	40 (43.0)	98 (25.4)*	168 (41.6)	< 0.001
APACHE II	14 (10, 20)	16 (12, 22)	14 (10, 19)	16 (11, 22)	0.742

*P value for the difference between this group and the other two groups was less than 0.0167

†*P* value for the difference between the pharmacological prophylaxis group and pharmacological combined mechanical prophylaxis group was less than 0.0167

*AECOPD* acute exacerbation of chronic obstructive pulmonary disease, *ARDS* acute respiratory distress syndrome, *BMI* body mass index, *IPA* invasive pulmonary aspergillosis, *OSAHS* obstructive sleep apnea-hypopnea syndrome

For patients who underwent VTE prophylaxis, the median age was 61 (IQR 48,70) years, the median BMI was 23.7 (IQR 20.8, 26.7) kg/m^2^, and 66.9% patients were male. The pharmacological combined with mechanical prophylaxis group had a higher proportion of acute respiratory distress syndrome (ARDS) and pneumonia than the other groups (*P* < 0.05). The number of patients with acute exacerbation of chronic obstructive pulmonary disease (AECOPD) and invasive pulmonary aspergillosis (IPA) in the pharmacological prophylaxis group was more than that in the other two groups (*P* < 0.05).

Patients in the mechanical prophylaxis group had lower hemoglobin, platelet, and fibrinogen levels and a higher urea nitrogen level than patients in the other two groups (*P* < 0.05). Patients in the pharmacological combined mechanical prophylaxis group had a shorter activated partial thromboplastin time and a higher C-reactive protein level than those in the pharmacological prophylaxis group patients (*P* < 0.05). The d-dimer level of the pharmacological prophylaxis group was significantly higher than that in the other two groups (*P* < 0.001) (Table 2). The number of patients with an intravascular tube and underwent invasive mechanical ventilation was greater in the pharmacological combined mechanical prophylaxis compared to other measures (*P* < 0.001) (Table 3).

**Table 2:** Laboratory tests of patients in different prophylaxis measures

	Overall (*N* = 884)	Mechanical prophylaxis group (*N* = 93)	Pharmacological prophylaxis group (*N* = 387)	Pharmacological combined mechanical prophylaxis group (*N* = 404)	*P*-value
Leukocyte (×10^9^/L) (IQR)	10.4 (6.7, 14.5)	10.4 (5.9, 14.5)	10.1 (6.7, 15.1)	10.7 (6.9, 14.3)	0.811
Hemoglobin (g/L) (IQR)	109 (90, 128)	94 (73, 114)*	111 (95, 129)	109 (90, 129)	< 0.001
Platelet (×10^9^/L) (IQR)	172 (118, 248)	144 (70, 225)*	175 (125, 248)	174 (120, 253)	0.006
D-Dimer (ng/ml) (IQR)	5.0 (1.8,35.2)	4.1 (1.6,35.2)	14.3 (2.3, 25.6)*	3.9 (1.7, 10.7)	< 0.001
Prothrombin time (s) (IQR)	12.9 (11.8, 14.2)	13.0 (11.7, 14.7)	12.4 (11.6, 13.8)*	13.2 (12.1, 14.4)	< 0.001
Fibrinogen (mg/dl) (IQR)	398.0 (279.1, 524.5)	392.9 (268.8, 537.2)	413.5 (392.2, 549.2)	415.5 (294.6, 587.1)	< 0.001
APTT (s) (IQR)	30.9 (25.7, 37.5)	31.0 (25.3, 39.5)	32.1 (27.1, 39.1)†	29.5 (24.9, 35.5)†	< 0.001
AST (U/L) (IQR)	37 (23, 66)	35 (20, 54)	36.0 (24,65)	39 (25,69)	0.080
ALT (U/L) (IQR)	25 (15, 46)	22 (13, 40)	24 (15,42)	28 (16,53)	0.034
Albumin (g/L) (IQR)	29.0 (25.4, 32.5)	28.6 (24.1, 32.5)	28.1 (24.9, 31.8)†	29.7 (26.5, 33.0)†	0.001
Total bilirubin (μmol/L) (IQR)	11.5 (8.2, 17.0)	10.6 (8.2, 19.6)	11.4 (8.2, 15.9)	11.6 (8.4, 17.8)	0.578
Direct bilirubin (μmol/L) (IQR)	5.3 (3.4, 8.6)	5.0 (3.4, 8.5)	4.9 (3.2, 7.6)†	5.7 (3.4, 9.5)†	0.048
Creatinine (μmol/L) (IQR)	73.5 (51.3, 119.6)	83.7 (57.4, 173.9)	74.4 (51.6, 116.7)	69.8 (50.3, 120.4)	0.058
BUN (mmol/L) (IQR)	8.1 (5.6, 13.4)	10.6 (6.8, 16.1)*	8.0 (5.3, 13.4)	7.8 (5.6, 12.4)	0.005
ESR (mm/h) (IQR)	23.0 (10.0, 40.0)	18.5 (5.5, 32.0)¶	20.0 (10.0, 35.0)	28.0 (11.0, 43.5)¶	0.009
C-reactive protein (mg/L) (IQR)	9.6 (3.0, 16.5)	7.9 (2.7, 16.2)	8.8 (2.2, 14.7)†	10.8 (4.6, 19.4)†	< 0.001

*P value for the difference between this group and the other two groups was less than 0.0167

†*P* value for the difference between the pharmacological prophylaxis group and the pharmacological combined mechanical prophylaxis group was less than 0.0167

¶*P* value for the difference between the mechanical prophylaxis group and the pharmacological combined mechanical prophylaxis group was less than 0.0167

*APTT* activated partial thromboplastin time, *AST* aspartate aminotransferase, *ALT* glutamic-pyruvic transaminase, *BUN* blood urea nitrogen, *ESR* erythrocyte sedimentation rate

**Table 3:** Treatments for different prophylaxis groups during thromboprophylaxis

	Overall (*N* = 884)	Mechanical prophylaxis group (*N* = 93)	Pharmacological prophylaxis group (*N* = 387)	Pharmacological combined mechanical prophylaxis group (*N* = 404)	*P* value
CRRT, n (%)	181 (20.5)	7 (7.5)	67 (17.3)	107 (26.5)	< 0.001^¶^
ECMO, n (%)	112 (12.7)	6 (6.5)	47 (12.1)	59 (14.6)	0.095
Intravascular tube, n (%)	619 (70.0)	60 (64.5)	211 (54.5)	348 (86.1)*	< 0.001
Deep vein catheterization, n (%)	253 (28.6)	21 (22.6)	103 (26.6)	129 (31.9)	0.101
PICC, n (%)	23 (2.6)	5 (5.4)	9 (2.3)	9 (2.2)	0.219
Artery cannulation, n (%)	572 (64.7)	52 (55.9)	183 (47.3)	337 (83.4)*	< 0.001
Swan-Ganz catheter, n (%)	31 (3.5)	1 (1.1)	13 (3.4)	17 (4.2)	0.359
Invasive mechanical ventilation, n (%)	615 (69.6)	57 (61.3)	231 (59.7)	327 (80.9)*	< 0.001
Non-invasive mechanical ventilation, n (%)	367 (41.5)	30 (32.3)†	176 (45.5)†	161 (39.9)	0.044
Artificial airway, n (%)	609 (68.9)	57 (61.3)	228 (58.9)	324 (80.2)*	< 0.001
Intubation of the trachea, n (%)	573 (64.8)	54 (58.1)	217 (56.1)	302 (74.8)*	< 0.001
Tracheotomy, n (%)	178 (20.1)	11 (11.8)	51 (13.2)	116 (28.7)*	< 0.001
Blood component transfusion, n (%)	345 (39.0)	49 (52.7)	118 (30.5)*	178 (44.1)	< 0.001

*P value for difference between this group and the other two groups were less than 0.0167

†*P* value for difference between mechanical prophylaxis group and the pharmacological prophylaxis group was less than 0.0167

¶*P* value for difference between any two groups was less than 0.0167

*CRRT* continuous renal replacement treatment, *ECMO* extracorporeal membrane oxygenation, *PICC* peripherally inserted central catheter

### VTE event of different prophylaxis measures

The total proportion of VTE events was 14.3% (126/884) for patients who underwent VTE prophylaxis, of which 0.1% (1/884) had PTE, 2.0% (18/884) had proximal DVT, and 12.1% (107/884) had isolated distal deep vein thrombosis (IDDVT). The proportions of proximal DVT or IDDVT among different prophylaxis groups were not significantly different (Table 4).

**Table 4:** Outcomes for different prophylaxis

	Overall (*N* = 884)	Mechanical prophylaxis group (*N* = 93)	Pharmacological prophylaxis group (*N* = 387)	Pharmacological combined mechanical prophylaxis group (*N* = 404)	*P* value
VTE, n (%)	126 (14.3)	13 (14.0)	47 (12.1)	66 (16.3)	0.240
PTE, n (%)	1 (0.1)	0 (0)	1 (0.3)	0 (0.0)	–
DVT, n (%)	125 (14.1)	13 (14.0)	46 (11.9)	66 (16.3)	0.201
Proximal DVT, n (%)	18 (2.0)	1 (1.1)	6 (1.6)	11 (2.7)	0.452
Proximal DVT alone, n (%)	9 (1.0)	1 (1.1)	3 (0.8)	5 (1.2)	–
Proximal and distal DVT, n (%)	9 (1.0)	0 (0)	3 (0.8)	6 (1.5)	–
IDDVT, n (%)	107 (12.1)	12 (12.9)	40 (10.3)	55 (13.6)	0.369
Bleeding events, n (%)	143 (16.2)	13 (14.0)	67 (17.3)	63 (15.6)	0.683
Major bleeding events, n (%)	83 (9.4)	6 (6.5)	38 (9.8)	39 (9.7)	0.596
Anticoagulant-related thrombocytopenia, n (%)	5 (0.6)	0 (0.0)	4 (1.0)	1 (0.2)	0.331
Mortality during RICU hospitalization, n (%)	256 (29.0)	22 (23.7)	124 (32.0)	110 (27.2)	0.167
LOS in RICU (days, IQR)	12 (7, 20)	10 (6, 15)	10.0 (6, 19)	14.0 (9, 25)*	< 0.001

*P values for difference between this group and the other two groups were less than 0.0167

LOS, length of stay; RICU, respiratory intensive care unit; VTE, venous thromboembolism; PTE, pulmonary thromboembolism; DVT, deep vein thrombosis; IDDVT, isolated distal deep vein thrombosis

### Bleeding and thrombocytopenia event of different prophylaxis measures

About 16.2% (143/884) patients had bleeding events under the VTE prophylaxis protocol during the ICU stay, and 9.4% (83/884) of them were major bleeding events (Table 4). There were no significant differences in the proportion of bleeding events in different prophylaxis groups (*P* = 0.683). During thromboprophylaxis, five patients had anticoagulant-related thrombocytopenia, with no significant difference among different prophylaxis groups.

### ICU mortality and length of ICU stay

Mortality for patients who underwent VTE prophylaxis protocol was 29.0% (256/884), but there was no significant difference between different prophylaxis groups (*P* = 0.167) (Table 4). For patients with VTE, the mortality rate was 34.1% (43/126). There was no difference in mortality between patients whether or not they experienced VTE events during their ICU stay (*P* = 0.169). The LOS in the ICU of patients in the pharmacological combined mechanical prophylaxis group was 14 (9, 25) days, which is significantly longer than that in the other two groups (*P* < 0.001). The duration of the ICU stay of patients with VTE events was 20 (12, 35) days, which is significantly longer than in patients without VTE events of 14 (8, 26) days, *P* < 0.001.

### Risk factors for VTE under the prophylaxis protocol

**Fig. 3:**
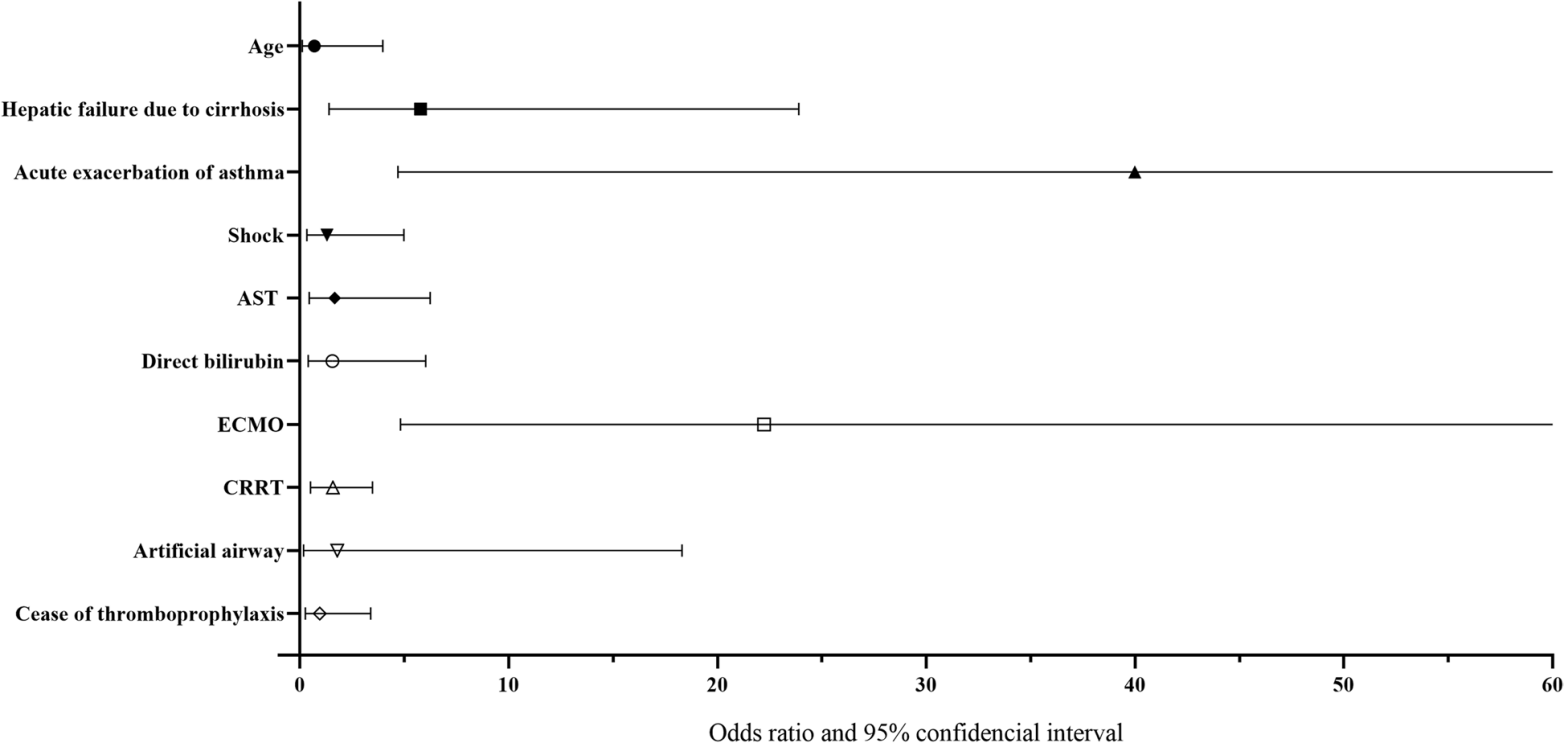
Forest plot of multivariate regression of proximal DVT. AST, aspartate aminotransferase; CRRT, continued renal replacement therapy; ECMO, extracorporeal membrane oxygenation

Multivariate logistic regression revealed hepatic failure due to cirrhosis (OR 5.789, 95% CI [1.402, 23.894], *P* = 0.015), acute asthma exacerbation (OR 39.999, 95% CI [4.704, 340.083], *P* = 0.001), and ECMO (OR 22.237, 95% CI [4.824, 102.502], *P* < 0.001) were independent risk factors of proximal DVT of patients in the ICU under the VTE prophylaxis protocol (Fig. 3 and Supplementary Table 2). For the aspect of IDDVT, artificial airway (OR 2.886, 95%CI [1.551, 5.372], *P* = 0.001) and duration of mechanical ventilation (OR 1.020, 95%CI [1.010, 1.029], *P* < 0.001) were the independent risk factors of patients under VTE prophylaxis (Supplementary Table 3).

## Discussion

The present study was the largest cohort study in mainland China focused on the VTE event proportion under a thromboprophylaxis protocol for critically ill patients in ICU. The thromboprophylaxis rate in this study was extremely high. However, there was still a certain occurrence of VTE events under this thromboprophylaxis protocol, albeit with a relatively lower proportion rate of PTE and proximal DVT. In this study, we found that the VTE events that happened were not associated with the increased risk of mortality among critically ill patients with thromboprophylaxis. Hepatic failure due to cirrhosis, acute exacerbation of asthma, and ECMO were the independent risk factors of proximal DVT despite being under the thromboprophylaxis protocol. Nevertheless, about 16.2% of the patients had bleeding events under this prophylaxis protocol, which should be paid more attention to during implementation.

Critically ill patients generally have more than one VTE risk factor [25]; therefore, thromboprophylaxis is recommended for such patients in many sets of guidelines [5, 6, 26]. The PROF-ETEV study from Spain found that about 41% of critically ill patients were receiving an inappropriate prophylaxis [13]. Research from Australia showed that early thromboprophylaxis was used in 74% patients within 24 h of ICU admission [9]. An observation study has reported that with the increase of the medical staff’s awareness of VTE prophylaxis in China, the VTE prophylaxis rate in ICUs was about 90.1% [27]. In the present study, the thromboprophylaxis rate in the ICU was 93.7%, which is relatively higher than that mentioned in previous literature.

A multi-center study showed that proximal DVT occurred in 5.1% of critically ill patients receiving LMWH [28]. Hamada et al. found that the prevalence of VTE was still 30.7% despite the well-driven thromboprophylaxis protocol in critically ill trauma patients [29]. A pooled analysis of two prospective cohort studies discovered that 2.2% of the critically ill patients receiving contemporary thrombosis prophylaxis developed PTE with or without DVT [10]. In this study, all the critically ill patients received thromboprophylaxis protocol once admitted to the ICU. The specific thromboprophylaxis measure selection should be done according to the bleeding risk and whether there exists an extremely high risk of VTE. We found that the proportion of proximal DVT and PTE was lower than that in the existing literature reported under this protocol. Therefore, this thromboprophylaxis protocol seemed to be more effective, which may indicate its good application prospects.

From the thromboprophylaxis measure distribution characteristics reported in this study, it could be found that nearly 90% of critically ill patients in a non-surgical ICU should receive at least pharmacological prophylaxis. There was no difference in the proportion of VTE events of patients between different thromboprophylaxis measures in this study. Thromboprophylaxis by mechanical alone is recommended for critical care patients at high risk of bleeding or active bleeding with contraindications to prophylactic anticoagulant agents because of the uncertain benefit of mechanical prophylaxis measures [5]. However, there has still been a lack of research on the thromboprophylaxis effect between pharmacological and mechanical prophylaxis measures. Although there were fewer patients in the mechanical prophylaxis group than in the other groups, this may hint at the equal effect of thromboprophylaxis according to the bleeding risk stratification in this protocol. It also indicates the rationality of this protocol.

Except for existing VTE risk assessment models, it is important to recognize the risk factor of VTE despite being under specific thromboprophylaxis. In this study, cirrhosis, asthma, and ECMO were the independent risk factors of proximal DVT under the thromboprophylaxis protocol. Some literature suggests that patients with cirrhosis have an increased risk of VTE [30]. The possible mechanisms are the reduction of anticoagulant factors, hyperactivation of thrombin, procoagulant activity caused by structural changes of fibrin, and platelet hyperreactivity in patients with cirrhosis. Impaired fibrinolysis might also be a mechanism for the increased risk of VTE in asthma patients [31], and this risk was higher in younger and more severe asthmatic patients [32]. With the increasing application of ECMO in clinical settings, ECMO-related VTE event has been found to range from 18.1 to 74% [33, 34]. The mechanism of thrombosis in ECMO may be not only with the local endothelial injury but also the difficulty in management of anticoagulation of ECMO.

In the present study, thromboprophylaxis protocol could effectively decrease the occurrence of proximal DVT and PTE, but 12.1% of the patients still acquired asymptomatic IDDVT. Currently, there is still controversy regarding the clinical benefit of treatment and long-term prognosis of IDDVT [35, 36], because there are very little data available on critically ill patients. We found that the presence of an artificial airway and the duration of mechanical ventilation were independent risk factors of IDDVT. This might be because early mobilization was limited by long time mechanical ventilation, which caused the muscle group of distal lower limb contraction weaken with slow venous reflux. Decreasing the proportion of IDDVT in ICU patients might be another important research topic in the future.

In this study, 9.4% of the patients received anticoagulation agents for thromboprophylaxis and suffered major bleeding events. The proportion of major bleeding events did not differ between different thromboprophylaxis measures. However, the prevalence of major bleeding in this study was higher than that in other LMWH-related studies [16]. A systematic review and meta-analysis showed that major bleeding did not appear to be significantly influenced by heparin thromboprophylaxis in the ICU setting [15]. Another high-risk factor for bleeding seems to be patients on ECMO [37]. Indeed, patients who underwent ECMO are with high risk of bleeding and thrombosis at same time. Meanwhile, when patients on ECMO were excluded from analysis in this study, the proportion of major bleeding decreased to 6.2%, which is in line with existing reports.

There were some limitations in this study. First, this was a single-center cohort study, which might induce an unavoidable selection bias. Second, independent risk factors of major bleeding under this thromboprophylaxis protocol were not analyzed, which should be the next important work of this cohort study. Third, during the present study, thromboprophylaxis measures transitioned in different arms according to the risk of bleeding and VTE changes during ICU stay. It had been difficulty in grouping patients exactly, which might affect the power of the result. We tried to minimize these interfere by grouped the patients with the longest duration of the prophylaxis measures before end-point events. Lastly, this study was conducted in a respiratory ICU, and the result could only be spread among non-surgical critically ill patients. Although this thromboprophylaxis protocol was carried out based on the assessment of bleeding and VTE risks, its safety and effect should be further explored, not only in an enlarged sample size study but also in different ICUs and regions.

## Conclusion

The thromboprophylaxis protocol for critically ill patients drawn up based on guidelines was practicable in the ICU, which with the potential to help reduce the proximal VTE and PTE event proportions. However, this protocol may have an unsatisfactory effect in some special patients. Therefore, it is important to recognize the risk factors of VTE events happening under the thromboprophylaxis protocol. Early intervention or strengthening of prophylaxis measures may help to reduce the risk of VTE in such patients. Meanwhile, anticoagulation agents related to major bleeding should be monitored while implementing this protocol. Further study should focus on these factors to perfect the thromboprophylaxis protocol in the future.

## Supplementary Information

**Additional file 1: Supplementary Table 1**. Reasons patients did not undergo the thromboprophylaxis protocol. **Supplementary Table 2**. Univariate and Multivariate Analysis of Potential Risk Factors for Proximal DVT. **Supplementary Table 3**. Univariate and multivariate analysis of potential risk factors for IDDVT.

## Data Availability

The datasets used and analysed during the current study are available from the corresponding author on reasonable request.

## Abbreviations

AECOPD: Acute exacerbation of chronic obstructive pulmonary disease
APTT: Activated partial thromboplastin time
ARDS: Acute respiratory distress syndrome
CRRT: Continuous renal replacement therapy
CUS: Compression ultrasonography
DVT: Deep vein thrombosis
ECMO: Extracorporeal membrane oxygenation
GSC: Graduated compression stocking
HIT: Heparin-induced thrombocytopenia
ICU: Intensive care unit
IDDVT: Isolated distal deep vein thrombosis
IPA: Invasive pulmonary aspergillosis
IPC: Intermittent pneumatic compression
IQR: Interquartile range
LOS: Length of stay
LMWH: Low molecular weight heparin
PTE: Pulmonary thromboembolism
UFH: Unfractionated heparin
VTE: Venous thromboembolism
