# Supplementary material for: Modern thromboprophylaxis protocol based on guidelines applied in a respiratory intensive care unit: a single-center prospective cohort study
Source: Thromb J. 2022 Dec 12;20:76. doi: 10.1186/s12959-022-00439-2 (PMC9746213; doi:10.1186/s12959-022-00439-2)
Supplement: Supplementary file 1 — Additional file 1: Supplementary Table 1. Reasons patients did not undergo the thromboprophylaxis protocol. Supplementary Table 2. Univariate and Multivariate Analysis of Potential Risk Factors for Proximal DVT. Supplementary Table 3. Univariate and multivariate analysis of potential risk factors for IDDVT. [file 12959_2022_439_MOESM1_ESM.docx]

Supplementary Table 1. Reasons patients did not undergo the thromboprophylaxis protocol.

|  | No VTE prophylaxis  N = 59 |
| --- | --- |
| High bleeding risk, medical staff did not implement mechanical prophylaxis | 21 |
| Active bleeding, medical staff did not implement mechanical prophylaxis | 15 |
| No reason | 9 |
| Anticoagulant contraindication, medical staff did not implement mechanical prophylaxis | 6 |
| Mechanical prophylaxis contraindication | 3 |
| Refusal | 3 |
| Menstruation, medical staff did not implement any prophylaxis measures | 2 |

Table 2. Univariate and Multivariate Analysis of Potential Risk Factors for Proximal DVT.

|  |  | Univariate analysis | | |  | Multivariate analysis | | |
| --- | --- | --- | --- | --- | --- | --- | --- | --- |
|  |  | Odds ratio | 95% CI | p-value |  | Odds ratio | 95% CI | p-value |
| Age |  | 0.29 | 0.07, 1.28 | 0.102 |  |  |  |  |
| Hepatic failure due to cirrhosis |  | 5.65 | 1.79, 17.82 | 0.003 |  | 5.79 | 1.40, 23.89 | 0.015 |
| Acute exacerbation of asthma |  | 6.67 | 1.41, 31.46 | 0.017 |  | 40.00 | 4.70, 340.08 | 0.001 |
| Shock |  | 3.58 | 1.15, 11.12 | 0.028 |  |  |  |  |
| Post-surgery |  | 2.42 | 0.78, 7.44 | 0.124 |  |  |  |  |
| AST |  | 2.68 | 0.94, 7.67 | 0.066 |  |  |  |  |
| Direct bilirubin |  | 3.25 | 1.25, 8.50 | 0.016 |  |  |  |  |
| Endovascular catheters |  | 7.91 | 1.05, 59.54 | 0.045 |  |  |  |  |
| ECMO |  | 29.69 | 9.66, 91.27 | 0.000 |  | 22.24 | 4.82, 102.50 | <0.001 |
| CRRT |  | 4.51 | 1.80, 11.27 | 0.001 |  |  |  |  |
| Invasive mechanical ventilation |  | 8.08 | 1.07, 60.84 | 0.043 |  |  |  |  |
| Non-invasive mechanical ventilation |  | 0.37 | 0.12, 1.12 | 0.078 |  |  |  |  |
| Artificial airway |  | 8.35 | 1.11, 62.83 | 0.039 |  |  |  |  |
| Vasopressor |  | 3.80 | 1.36, 10.64 | 0.011 |  |  |  |  |
| Cease of thromboprophylaxis |  | 2.45 | 0.87, 2.45 | 0.091 |  |  |  |  |

AST, aspartate aminotransferase; CRRT, Continuous Renal Replacement Treatment; DVT, Deep Vein Thrombosis; ECMO, Extracorporeal membrane oxygenation; PTE, Pulmonary Thromboembolism.

Supplementary Table 3. Univariate and multivariate analysis of potential risk factors for IDDVT.

|  |  | Univariate analysis | | |  | Multivariate analysis | | |
| --- | --- | --- | --- | --- | --- | --- | --- | --- |
|  |  | Odds ratio | 95% CI | p-value |  | Odds ratio | 95% CI | p-value |
| Padua score |  | 1.52 | 1.01, 2.30 | 0.045 |  |  |  |  |
| Pneumonia |  | 2.05 | 1.25, 3.35 | 0.004 |  |  |  |  |
| ARDS |  | 1.82 | 1.20, 2.74 | 0.005 |  |  |  |  |
| AECOPD |  | 0.51 | 0.23, 1.13 | 0.096 |  |  |  |  |
| Shock |  | 1.76 | 0.91, 3.42 | 0.094 |  |  |  |  |
| Cancer |  | 1.78 | 0.89, 3.55 | 0.102 |  |  |  |  |
| Platelet |  | 0.63 | 0.41, 0.97 | 0.034 |  |  |  |  |
| Endovascular catheters |  | 1.65 | 1.02, 2.69 | 0.043 |  |  |  |  |
| PICC |  | 2.77 | 1.05, 7.29 | 0.039 |  |  |  |  |
| ECMO |  | 1.84 | 1.09, 3.11 | 0.023 |  |  |  |  |
| Vasopressor |  | 1.98 | 1.31, 2.98 | 0.001 |  |  |  |  |
| Artificial airway |  | 3.36 | 1.88, 6.01 | 0.000 |  | 2.89 | 1.55, 5.37 | 0.001 |
| Blood transfusion |  | 2.11 | 1.40, 3.17 | 0.000 |  |  |  |  |
| Cease of thromboprophylaxis |  | 1.65 | 0.97, 2.81 | 0.065 |  |  |  |  |
| Proton pump inhibitor |  | 1.76 | 1.06, 2.94 | 0.030 |  |  |  |  |
| Duration of mechanical ventilation |  | 1.012 | 1.003, 1.020 | 0.006 |  | 1.020 | 1.010, 1.029 | <0.001 |

ARDS, acute respiratory distress syndrome; AECOPD, Acute exacerbation of chronic obstructive pulmonary disease; ECMO, Extracorporeal membrane oxygenation; ICU, intensive care unit; IDDVT, isolated distal deep vein thrombosis; PICC, Peripherally Inserted Central Catheter.
